# Protocol for the Birth Asphyxia in African Newborns (Baby BRAiN) Study: a Neonatal Encephalopathy Feasibility Cohort Study

**DOI:** 10.12688/gatesopenres.13557.1

**Published:** 2022-03-03

**Authors:** Carol Nanyunja, Samantha Sadoo, Ivan Mambule, Sean R Mathieson, Moffat Nyirenda, Emily L Webb, J Mugalu, Nicola J Robertson, A Nabawanuka, Guillaume Gilbert, J Bwambale, Kathryn Martinello, Alan Bainbridge, Samson Lubowa, Latha Srinivasan, H Ssebombo, Cathy Morgan, Cornelia Hagmann, Frances M Cowan, Kirsty Le Doare, Pia Wintermark, Michael Kawooya, Geraldine B Boylan, Annettee Nakimuli, Cally J Tann

**Affiliations:** 1 MRC/UVRI & LSHTM Uganda Research Unit, Entebbe, Uganda; 2 London School of Hygiene and Tropical Medicine, London, WC1E 7HT, UK; 3 INFANT Research Centre, University College Cork, Cork, Ireland; 4 Kawempe National Referral Hospital, Kampala, UK; 5 University College London, London, UK; 6 University of Edinburgh, Edinburgh, UK; 7 Kampala MRI Centre, Kampala, Uganda; 8 MRC Clinical Science, Philips Healthcare, Ontario, Canada; 9 Great Ormond Street Hospital, London, WC1N 3JH, UK; 10 Makarere University, Kampala, Uganda; 11 Cerebral Palsy Alliance Research Institute, University of Sydney, Sydney, Australia; 12 Children’s University Hospital of Zurich, Zurich, Switzerland; 13 Imperial College London, London, UK; 14 St George's, University of London, London, UK; 15 McGill University, Montreal, Canada; 16 Ernest Cook Ultrasound Research and Education Institute (ECUREI), Kampala, Uganda

**Keywords:** Neonatal Encephalopathy, Magnetic Resonance Imaging, Magnetic Resonance Spectroscopy, Electroencephalography, outcomes, neurodevelopmental impairment, Uganda, Low- and Middle-Income Countries

## Abstract

**BACKGROUND:** Neonatal encephalopathy (NE) is a leading cause of child mortality worldwide and contributes substantially to stillbirths and long-term disability. Ninety-nine percent of deaths from NE occur in low-and-middle-income countries (LMICs). Whilst therapeutic hypothermia significantly improves outcomes in high-income countries, its safety and effectiveness in diverse LMIC contexts remains debated. Important differences in the aetiology, nature and timing of neonatal brain injury likely influence the effectiveness of postnatal interventions, including therapeutic hypothermia.

**METHODS: **This is a prospective pilot feasibility cohort study of neonates with NE conducted at Kawempe National Referral Hospital, Kampala, Uganda. Neurological investigations include continuous video electroencephalography (EEG) (days 1-4), serial cranial ultrasound imaging, and neonatal brain Magnetic Resonance Imaging and Spectroscopy (MRI/ MRS) (day 10-14). Neurodevelopmental follow-up will be continued to 18-24 months of age including Prechtl’s Assessment of General Movements, Bayley Scales of Infant Development, and a formal scored neurological examination. The primary outcome will be death and moderate-severe neurodevelopmental impairment at 18-24 months. Findings will be used to inform explorative science and larger trials, aiming to develop urgently needed neuroprotective and neurorestorative interventions for NE applicable for use in diverse settings.

**DISCUSSION: **The primary aims of the study are to assess the feasibility of establishing a facility-based cohort of children with NE in Uganda, to enhance our understanding of NE in a low-resource sub-Saharan African setting and provide infrastructure to conduct high-quality research on neuroprotective/ neurorestorative strategies to reduce death and disability from NE. Specific objectives are to establish a NE cohort, in order to 1) investigate the clinical course, aetiology, nature and timing of perinatal brain injury; 2) describe electrographic activity and quantify seizure burden and the relationship with adverse outcomes, and; 3) develop capacity for neonatal brain MRI/S and examine associations with early neurodevelopmental outcomes.

## Introduction

Globally, neonatal encephalopathy (NE) is a leading cause of under-5 child mortality and a significant contributor to stillbirths, morbidity and long-term disability ^1^. There are an estimated 1.15 million cases of NE each year, and 627,000 deaths from NE, of which 99% occur in low- and middle-income countries (LMICs) ^2^. More than 400,000 children each year develop neurodevelopmental impairment (NDI) after NE including cerebral palsy, seizure disorders, and visual, hearing, and cognitive impairments ^2^. Of these, 40% are estimated to reside in sub-Saharan Africa (SSA) where limited access to inclusive support services may exacerbate socioeconomic burden and impair quality of life ^2^. The UN Global Strategy for Women’s, Children’s and Adolescents’ Health (2016–2030) signals a shift in focus on the global agenda towards development and wellbeing, advocating that children should not only ‘survive’ but also ‘thrive’ ^3^.

NE is a clinical syndrome of brain dysfunction defined as a “disturbance of neurological function in the earliest days after birth in the term or near-term infant, manifested by difficulty initiating and maintaining respiration, depression of tone or reflexes, abnormal level of consciousness, and often by seizures” ^4^. In a recent Ugandan study, half of NE infants had clinical evidence of abnormal movements consistent with seizures ^5^; however, it is known that around two-thirds of seizures are not clinically apparent ^6^. Seizures can exacerbate neuronal injury by increasing cerebral metabolic demand, triggering release of excitatory neurotransmitters and causing cardiorespiratory instability. Seizure burden has a significant independent association with the severity of brain injury on MRI, and with NDI at 18–24 months ^7– 9^. Continuous electroencephalogram (EEG) is the gold standard investigation for seizure detection and background activity and provides assessment of severity of injury, prognostication, and evolution over time ^10^. However, EEG is often not available in LMICs and few studies in SSA involving EEG have been published to date.

Magnetic Resonance Imaging (MRI) brain is the gold standard imaging modality for assessment and prognostication of hypoxic-ischaemic encephalopathy (HIE) in high-income countries (HICs); however, it is not available in most LMIC settings. Intrapartum-related hypoxia ischaemia causes pathognomonic abnormalities in the basal ganglia, thalamus and posterior limb of the internal capsule (PLIC), as well as white matter changes ^11^. Conventional MRI (T1 and T2 weighted imaging) within the first two weeks after the insult is 98% sensitive and 76% specific for predicting long-term neurodevelopmental outcome after NE ^12^. Magnetic resonance spectroscopy (MRS) is not yet used routinely in clinical practice, but may serve as an early biomarker for brain injury and increase the predictive value of MRI. In the HIC setting, the Lactate/N-Acetylaspartate (Lac/NAA) ratio is highly specific (98%) for predicting long-term outcomes ^13^. A thalamic Lac/NAA ratio ≥0.39 accurately predicts poor motor, cognitive and language outcome at two years for neonates undergoing therapeutic hypothermia ^14^.

In HICs, therapeutic hypothermia (‘cooling’ the whole body to a core temperature of 33.5°C for 72 hours, initiated within six hours of birth) is strongly evidenced to reduce death and disability after intrapartum-related hypoxia-ischaemia ^15^. However, its safety and effectiveness across diverse LMIC settings remains inconclusive ^16^. Differences in obstetric and neonatal care settings and in the aetiology, nature and timing of brain injury may all contribute; however, these differences have been poorly defined.

The Baby BRAiN Study aims to assess the feasibility of establishing a facility-based cohort of infants with NE in Uganda, to enhance our understanding of NE in a low-resource sub-Saharan African setting, and provide infrastructure to conduct future high-quality research on neuroprotective and neurorestorative strategies, with the ultimate aim of developing novel effective interventions for NE applicable for use in diverse settings.

### Objectives

The specific objectives of the study are to:

1. Establish a pilot NE cohort in Uganda to investigate the clinical course, nature and timing of perinatal brain injury and associations with adverse outcomes.

2. Describe electrographic brain activity and seizure burden amongst Ugandan neonates with NE, and their relationship with adverse outcomes.

3. Develop capacity for neonatal brain MRI/S amongst Ugandan neonates with NE, and associations with adverse outcomes.

## Methods

### Study design

This is a prospective feasibility facility-based cohort study of neonates with NE.

### Setting

Uganda is a low-income country situated in East Africa, ranking 176 ^th^ out of 193 countries for GDP per capita ^17^. The neonatal mortality rate is 19 deaths per 1000 live births ^1^. Uganda has a population of around 43 million, of whom 1.6 million live in the capital city Kampala.

The study is based at Kawempe National Referral Hospital (KNRH), the largest maternity facility in Kampala (the capital city of Uganda) receiving high-risk referrals from across the city, with around 21,000 deliveries each year. The incidence of NE is estimated to be 15–20 per 1000 live births, with 300–350 neonates with moderate-severe NE admitted each year. Routine care includes continuous positive airway pressure (CPAP) ventilation, intravenous fluids including glucose, antibiotics, and anticonvulsant medication. Therapeutic hypothermia is not offered, consistent with other low-resource settings/ non-intensive care facilities.

### Participants

**Figure 1.**
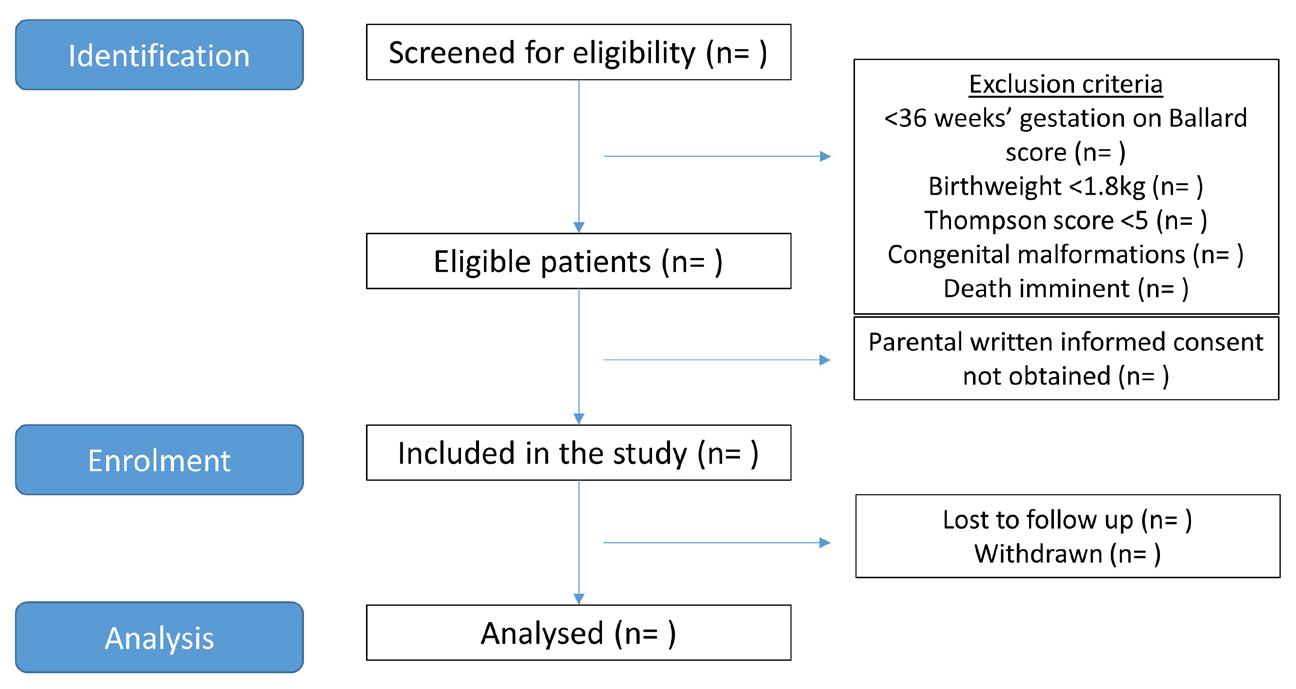
Flow of participants. Planned flow of participants through the study from screening to analysis.

Participants are term and near-term neonates with NE admitted to the neonatal unit at KNRH, Uganda. The planned flow of participants is presented in Figure 1.

***Eligibility screening.*** Neonates affected by intrapartum-related hypoxic-ischaemic insults are frequently born in poor condition requiring resuscitation after birth, and manifest clinical signs of encephalopathy shortly after birth. At KNRH all such infants are transferred to the neonatal unit for ongoing care, and referrals are also accepted from communities and district hospitals across Kampala.

**Figure 2.**
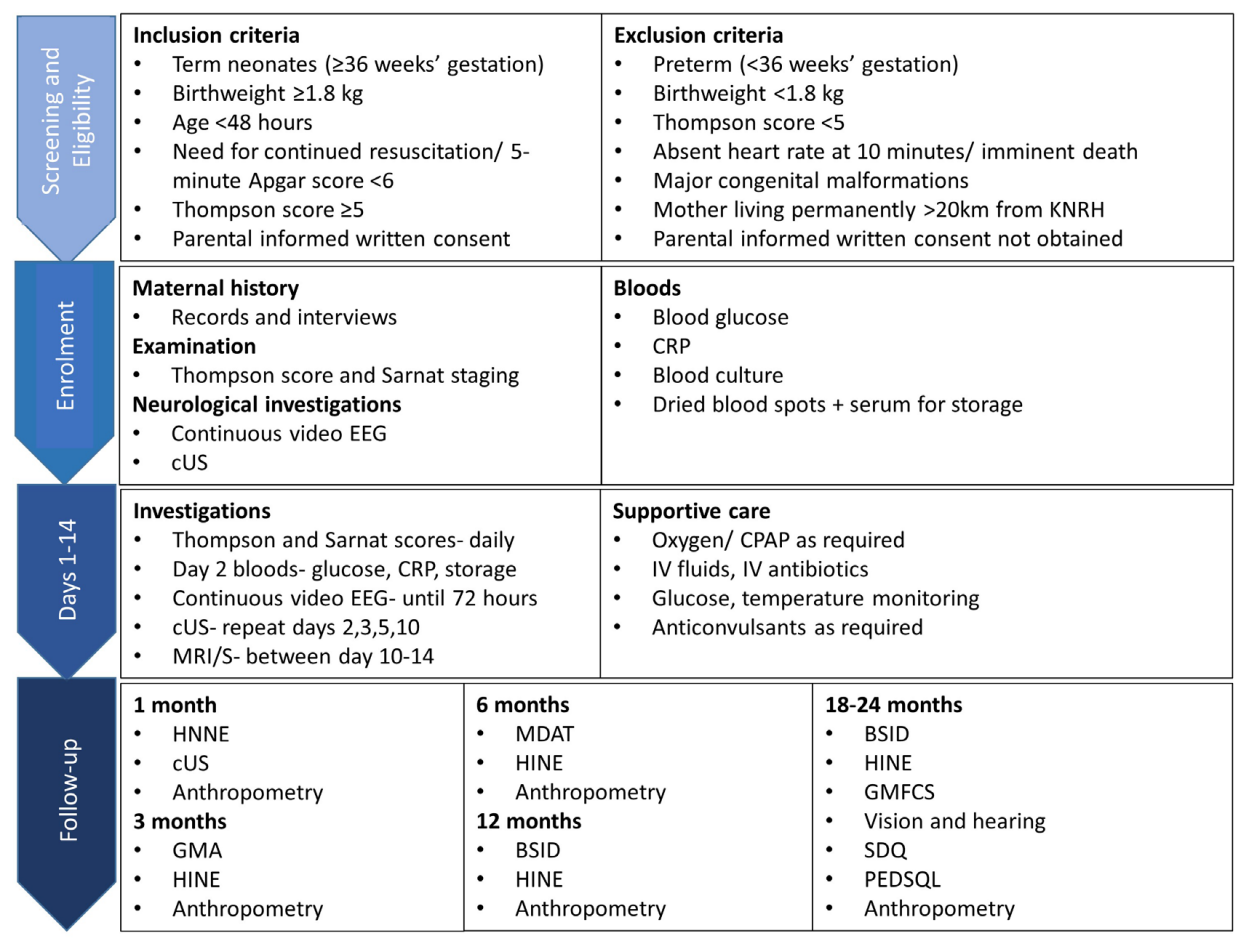
Study procedures and follow-up. Study procedures, including inclusion/ exclusion criteria, from screening to follow-up. Abbreviations: KNRH= Kawempe National Referral Hospital; EEG= Electroencephalogram; cUS= Cranial Ultrasound; CRP= C-Reactive Protein; EDTA= Ethylenediaminetetraacetic acid; MRI= Magnetic Resonance Imaging; MRS= Magnetic Resonance Spectroscopy; CPAP= Continuous Positive Airway Pressure; IV= Intravenous; HINE= Hammersmith Infant Neurological Examination; GMA= Prechtl’s Assessment of General Movements; MDAT= Malawi Development Assessment Tool; BSID-III= Bayley Scales of Infant and Toddler Development III; GMFCS= Gross Motor Function Classification System; PEDSQL= Pediatric Quality of Life Inventory; SDQ= Strengths and Difficulties Questionnaire.

On arrival to the neonatal unit, the neonate will be clinically assessed for the need for resuscitation/ stabilisation. Once clinically stable, they will be assessed for eligibility to enter the study according to the inclusion and exclusion criteria ( Figure 2). Trained study staff will confirm: the location of the mother’s permanent residence; Apgar score at five minutes of age; birth weight; gestational age using the Ballard score; absence of major congenital abnormalities on clinical examination; and complete a neurological examination to assign a Thompson score for evidence of moderate-severe NE.

***Consent.*** Written informed parental consent will be sought as soon as possible after a baby has been identified as eligible for the study. If the mother is unavailable or too ill to provide consent, informed consent will be obtained from the father. Both verbal and written explanations in English/ Luganda will be provided. Separate consent forms will be required for radiological images and video recordings.

### Clinical supportive care

All infants will be managed clinically according to standard practice at KNRH neonatal unit, supported by the Uganda Paediatric Association (UPA) guidelines. For all unwell neonates, including those presenting with encephalopathy, an intravenous cannula will be inserted and blood samples including at least 1mL of blood for blood cultures (BACTEC Peds Plus, NJ, USA) taken. Intravenous 10% dextrose and intravenous antibiotics will be commenced. Supplementary nasal cannula oxygen will be given according to oxygen saturations. Clinical seizures will be treated with first-line anticonvulsant intravenous Phenobarbitone, and second line treatment Diazepam if required.

### Data collection

All clinical procedures will be conducted by trained research personnel according to clear standard operating procedures. A diagram outlining study procedures is shown in Figure 2.

***Baseline data collection.*** After written informed parental consent is obtained, baseline data will be collected on pre-conception, antepartum and intrapartum exposures. This will include sociodemographic, medical, and obstetric history. Information will be obtained from structured maternal interviews, antenatal and hospital clinical records, and documented on standardised data collection forms. Baseline anthropometric measurements will be measured (birth weight, length, occipito-frontal circumference).

Relevant clinical data will be collected including temperature, respiratory support, antibiotic administration, suspected or culture positive sepsis, anticonvulsant medication administration, age at discharge, method of feeding at discharge, and death before discharge.

***Neurological examination: Thompson score and modified Sarnat staging.*** The Thompson score was first developed in a South African population in 1997, and has a maximum score of 22 based on nine neurological signs ^10, 18^. The modified Sarnat and Sarnat staging system is widely used in HICs for infants with NE to attribute a grade of mild, moderate or severe, based on clinical parameters including level of consciousness, tone and reflexes, seizures and duration of symptoms ^19^.

Neurological examinations will be performed daily between days one-five. This reflects the evolution of encephalopathy after a hypoxic insult over several days, usually peaking on day three ^19^. The findings will be used to assign both a Thompson score and Sarnat stage. Overall severity of NE will be defined according to the highest score.

***Hypoglycaemia.*** Glucose will be checked at recruitment using the Accu-Chek Active glucometer (Roche Diagnostics GmbH, Mannheim, Germany). Hypoglycaemia will be defined as <2.6mmol/l; IV fluids will be given in response to a low glucose as per UPA guidelines, and further monitoring conducted.

***Clinical seizures.*** Clinical seizures based on abnormal movements (limb, facial or eye) noted by staff will be documented, including the duration of clinical seizures.

***Continuous video electroencephalography (EEG).*** To evaluate brain function and presence of seizures, multichannel video EEG will be continuously measured and recorded over days 1–4 using the portable Lifelines iEEG systems (Lifelines iEEG, UK).

EEG disposable surface electrodes (disposable AMBU (Copenhagen, Denmark) Neuroline cup electrodes)) will be applied to the scalp, located at F3, F4, C3, C4, T3, T4, O1, O2 and Cz, according to the international 10–20 system adjusted for neonates along with single channel electrocardiography and respiration monitoring if possible. Trained staff will regularly assess the quality of the recordings (i.e. impedance, video camera position); otherwise the screen will be obscured from view.

Each EEG recording will be retrospectively analysed by an experienced neonatal electrophysiologist, blind to all clinical information with the exception of gestational age at birth. The background pattern of each EEG will be classified according to Murray *et al.* at for 1 hour epochs at 12, 24, 48 and 72 hours of age and at the time of Thompson score, to accommodate the known evolution of EEG background activity in NE ^6^. All seizures in each recording will be counted and the duration of each seizure measured. An EEG seizure will be defined as a sudden repetitive stereotyped discharge lasting for at least 10 seconds on two or more EEG channels. Seizure burden will be defined as the number of electrographic seizure seconds in the total EEG recording. A neonatologist will retrospectively review the EEG video recordings for suspected clinical seizures. It is anticipated that some video may not be useable, due to poor lighting in the neonatal unit and/or poor quality images; these will be reported as non-diagnostic. The proportion of clinical seizures correlating with electrographic seizures, and proportion of electrographic seizures without clinical features will be calculated, and types of seizure semiology described.

***Cranial ultrasound (cUS).*** Cranial ultrasound imaging will be performed on days one, two, three, five, ten, and at one month using a hand portable ultrasound machine (Edge II, Sonosite). Standard cranial views (as previously described ^20^) and anterior cerebral artery Doppler measurements will be obtained. The scans will be reviewed for evidence of abnormal anatomy, cysts, calcifications, sub-optimal growth, and findings suggestive of established injury/haemorrhage or evolving injury predating delivery. The scans will also be assessed retrospectively in terms of HI injury, according to a graded scoring system ^20, 21^. The resistive index (RI) will be calculated from the Doppler measurements on early scans, an indicator of cerebral perfusion which can predict adverse outcomes) ^22^.

***Magnetic Resonance Imaging (MRI) and Spectroscopy (MRS).*** All neonates surviving to discharge will be eligible for brain imaging at the Kampala MRI Centre (KAMRIC). Neonates will be accompanied to KAMRIC with a caregiver and a healthcare professional, and scanned on a 1.5 Tesla scanner (Philips Achieva, Best, the Netherlands) using a standardised protocol (supplementary data). MRI/S imaging will be performed at a minimum of ten days of age once the baby is clinically stable and no further interventions are required. Prior to scanning, neonates will be wrapped, fed, and nested, and the scan performed whilst sleeping naturally; oral chloral hydrate may be administered if the neonate remains active. Ear defenders and continuous pulse oximetry monitoring will be used throughout the scan. The MRI protocol, adapted from that used in HIC settings ^14, 23^, will compromise T1- and T2-weighted images, diffusion weighted imaging (DWI), and diffusion tensor imaging (DTI). During the study, magnetic resonance arteriography (MRA), magnetic resonance venography (MRV), and susceptibility weighted imaging (SWI) will also be attempted to provide additional diagnostic and prognostic information. MR images will be reviewed and reported by a neuroradiologist and perinatal neurologist blind to all early clinical data except for gestational age. Scores will be assigned based on pattern and severity of injury using the National Institute of Child Health and Human Development (NICHD) ^24^ and the Rutherford ^25, 26^ MRI scoring systems. The MRS protocol is adapted from that used in HIC settings with a point-resolved spectroscopy sequence (PRESS) voxel in the left basal ganglia and thalamus, acquiring proton (1H) MRS using a long echo time (TE: 288ms) to acquire lactate/N acetyl aspartate (Lac/NAA) peak area ratios. Studies using phantoms have previously checked the uniformity of the magnet with those in HIC settings. Anonymised MR data will be transferred to University College London (London, UK) for post-processing and analysis.

***Laboratory work.*** At least 1mL of blood (neonates) or 5ml (adults) will be taken prior to the start of antibiotics for blood cultures (BACTEC, NJ, USA) to identify maternal and early neonatal sepsis in collaboration with the existing ‘PROGRESS’ Group B Streptococcus study at KNRH ^27^. A neonatal C-reactive protein (CRP) will be sent on days one and two. All women will have human immunodeficiency virus (HIV) status checked as part of routine care. Dried blood spots and venous blood will be collected for storage for future studies. For dried blood spots, four spots will be collected on GE Whatman 903 protein saver cards and stored in desiccant for 24 hours then frozen at -20C. Venous blood (0.75mls) will be collected in a K2-EDTA tube, centrifuged at ambient temperature, and plasma transferred into a sterile polypropylene tube taking care not to disturb the cell pellet. The tube will be stored at -80°C within 72 hours of blood draw (to prevent decay of cell-free DNA).

### Follow-up

The neurodevelopmental follow-up schedule is shown in Figure 2 and Table 1. Interrater reliability will be assessed for neurodevelopmental assessments.

**Table 1.** Schedule of neurodevelopmental assessments. The planned schedule of neurodevelopmental/growth follow-up assessments between 28 days to 24 months of age.

Age at follow-up	Neurodevelopment	Growth
**28–30 days**	HNNE	Weight, OFC
**3 months**	GMA, HINE	Weight, Height, OFC
**6 months**	MDAT, HINE	Weight, Height, OFC
**12 months**	BSID III, HINE	Weight, Height, OFC
**18–24 months**	BSID III, HINE Vision. hearing SDQ	Weight, Height, OFC

Abbreviations: HNNE= Hammersmith Neonatal Neurological Examination; OFC= Occipito-Frontal Circumference; GMA= Prechtl’s Assessment of General Movements; HINE= Hammersmith Infant Neurological Examination; SDQ= Strengths and Difficulties Questionnaire

The Hammersmith Neonatal Neurological Examination (HNNE) is a practical and easy to perform examination encompassing 34 items assessing tone, motor patterns, observation of spontaneous movements, reflexes, visual and auditory attention, and behaviour ^28^. It has been used in different clinical groups of term and preterm infants in the neonatal period and has been shown to correlate with MRI findings amongst infants with HIE ^29^, and has been used by us previously in Uganda ^30^.

Prechtl’s Assessment of General Movements (GMA) is a non-invasive 3-minute observational assessment of spontaneously generated movements performed using video while the infant is in quiet wakefulness ^31^. In high risk (preterm and NE term) infants, absent fidgety movements at 3–4 months post term age have a 98% sensitivity and 94% specificity for predicting cerebral palsy at 1 year ^32^, and strongly correlate with MRI abnormalities ^33, 34^. All videos will be independently assessed by an advanced trained clinician blind to all early clinical data except gestational age.

The Hammersmith Infant Neurological Examination (HINE) is a standardised scorable tool that has been validated as a predictor of motor outcome in different cohorts, and has been used previously in children with and without NE in Uganda ^35, 36^. After 5 months' corrected age, the HINE has been found to be the most predictive tool for cerebral palsy (90% sensitivity) ^37^. A score of ≥67 at 9–14 months has been shown to be predictive of independent walking at 2 years ^38^.

The Malawi Development Assessment Tool (MDAT) is a developmental screening tool validated in the African setting ^39^ with good reliability, validity and sensitivity for the identification of developmental disabilities across the four developmental domains (gross motor, fine motor, language, social).

Comprehensive assessment of child development will be conducted at 12 and 18–24 months of age using the Bayley Scales of Infant Development III (BSID-III), a widely used tool that assesses cognitive, motor and language development up to 48 months of age ^40^. Cerebral palsy (CP) will be diagnosed from the neurological examination and classified according to the Surveillance of Cerebral Palsy in Europe hierarchical classification ^41^. The severity of cerebral palsy will be classified using the Gross Motor Function Classification System for Cerebral Palsy (GMFCS) ^42^. All neurodevelopmental assessments will be performed by trained study staff in the study’s outpatient clinic room to minimise distraction. Early clinical data will not be made available to the assessor at the time of assessment.

Vision and hearing will be assessed according to HINE standardised procedures at 18 months ^43^. Assessment of vision comprises examining for intermittent or continuous deviation of the eyes or abnormal movements, and ability to fix and follow on a clear black/white target. Hearing assessment includes testing reaction to a stimulus (a rattle) held behind a visual range on each side. Severe visual/ hearing impairment will be defined as a score of <1; unable to follow a visual target or not responding to an auditory stimulus, respectively ^43^.

Family quality of life will be assessed using the Pediatric Quality of Life Family Impact module (PedsQL) ^44^, a structured interview previously used in Ugandan studies ^45^, which assesses caregivers’ physical, social, emotional, cognitive, wellbeing, communication, daily activities and level of worry. The Strengths and Difficulties Questionnaire (SDQ) is widely used to assess child mental health used previously in the Africa setting ^46^. Both assessments will be translated into the local language (Luganda) and completed by the participant’s caregiver at the 18–24 month follow-up.

Child anthropometry will be assessed using weight, length/height, occipito-frontal head circumference (OFC) and mid-upper arm circumference (MUAC). OFC and MUAC will be measured using a paper tape measure, weight using SECA336 electronic scales, and height using a SECA measure mat in the supine position. Wasting will be defined as moderate (weight-for-age z-score <-2 and/or MUAC <125mm), or severe (weight-for-age z-score <-3 and/or MUAC <115mm) ^47^.

### Outcomes

Adverse outcome after NE will be defined as death or moderate-severe NDI at 18–24 months. Severe NDI will be defined as a BSID-III cognitive composite score <70, BSID-III motor composite score <70, presence of cerebral palsy GMFCS levels 3–5, blindness, or profound hearing loss. Moderate NDI will be defined as a cognitive or motor BSID-III score 70–84, cerebral palsy GMFCS level 2, a seizure disorder, or hearing impairment.

### Data management

Data will be recorded and managed through REDCap (Research Electronic Data Capture) and hosted by MRC/UVRI & LSHTM Uganda Research Unit in Entebbe, Uganda. REDCap includes in-built access management and audit trail functions to track changes to data, implement role assignment, and restrict unauthorised access. Data from REDCap will be downloaded and stored on secure password-protected institutional servers at LSHTM, for analysis. Internally developed Stata verification/ cleaning do-files will be run to identify data that are missing, inconsistent, or out-of-range. The study coordinator will perform clinical quality checks to identify potential errors not captured in the automated verification process.

Imaging (MRI/S and cUS) data will be transferred from the machine in DICOM format, with the removal of patient identifying information during this process. The images can then be opened in DICOM format using the Osirix imaging software and interpreted blind. Video EEG data recorded on the Lifelines iEEG system will be uploaded to a secure cloud-based server (managed by Lifelines iEEG, UK/Kvikna Medical, Iceland), which can then be accessed for later analysis. Video recordings for GMA and HINE assessments will be stored securely at the MRC/UVRI data centre.

### Sample size

A purposive sample size of a minimum of 70 neonates will be sequentially recruited to the NE cohort has been chosen for this pilot feasibility study. This was decided pragmatically, considering both the funding period, and the incidence of NE at KNRH. However, restriction on recruitment to research studies during the Covid-19 pandemic limited recruitment to 51 infants.

### Data analysis

Demographic factors, clinical characteristics, seizure burden, neurological examination, GMA, cUS scores, MRI scores (NICHD and Rutherford as stated above ^24– 26^), and outcome data, will be summarised with counts (percentages) for categorical variables, mean (standard deviation [SD]) for normally distributed continuous variables or median (interquartile or entire range) for other continuous variables. The electrographic seizure burden and seizures recognised by bedside staff, and the proportion evident on retrospective video review will be reported as percentages. Pearson’s correlation will also be used to assess agreement between seizure burden detection by the various methods (EEG, bedside staff and video EEG review by neonatologist) and hourly and total seizure burden will be calculated. Spearman’s correlation will compare EEG grade and Thompson Score from day 2–5. The feasibility and acceptability of GMA will also be evaluated.

Relationships between exposure variables (NE severity as defined by Thompson and Sarnat scores, clinical seizures, electrographic seizures and background activity, hypoglycaemia, sepsis as defined by blood culture results, cUS scores, MRI scores) and adverse outcomes will be assessed using multivariable regression models, adjusting for potential confounding factors, and reported using risk ratios.

The feasibility of MR imaging will be evaluated by the proportion of scans performed during the planned time period, implementing the planned protocol, and completeness and diagnostic quality of the images. Acceptability will be assessed by the proportion of participants with parental consent receiving MR imaging. MRI findings will be reported descriptively, and the severity of the pattern of brain injury will be scored according to the Rutherford ^26, 48^, and National Institute of Child Health and Human Development (NICHD) ^49^ criteria. Associations between MRI/S findings and clinical scores, and adverse outcomes, will be examined. MRS will be analysed using the TARQUIN analysis package in line with the analysis method in HIC settings, and the ratio of BGT Lac/NAA calculated. Prognostic accuracy of biomarkers in predicting adverse outcomes will be assessed using receiver operating characteristic (ROC) curves and linear regression.

### Ethics

***Approval.*** Hospital, institutional and national approvals will be sought from the ethics committees of Uganda Virus Research Institute (UVRI); London School of Hygiene & Tropical Medicine (LSHTM); Ugandan National Committee of Science & Technology (UNCST); and the Ugandan President’s Office.

***Adverse events.*** UNCST national guidance will be followed throughout the study ^50^. Any adverse events during the study period, whether attributed to the study or not, will be reported promptly to the principal investigator and the senior doctor on the neonatal unit at the time of the event. Any serious adverse event will be reported to the Institutional Review Board of UVRI within 48 hours, and appropriate documenting processes completed.

### Dissemination

The study will be written up for publication in a peer-reviewed open access journal, and abstracts will be submitted for presentation at relevant international conferences. The main findings will be launched with a press release from MRC/UVRI, MARCH Centre at LSHTM, and on social media in Uganda, UK and globally. Findings will be shared with key stakeholders in Uganda including; Uganda National Neonatal Steering Committee (committee meeting presentation); Uganda Paediatric Association (annual conference presentation); KNRH (Department of Paediatrics, Department of Obstetrics and Gynaecology, KNRH Administration); and study participants, through local community leaders and at MRC/UVRI & LSHTM Uganda Research Unit open days to which participating families and the local community will be invited. 

### Study status

The Baby BRAiN Study commenced on 18 ^th^ October 2019 and is ongoing. Due to the Covid-19 pandemic, recruitment was paused on 17 ^th^ March 2020 and restarted on 19 ^th^ July 2020, and the sample size restricted to 51 participants. Neurodevelopmental follow-up of the cohort is ongoing and will be completed in April 2022.

## Discussion

### Significance and potential impact

With a lack of evidence to date supporting the safety or efficacy of therapeutic hypothermia in LMICs, there is an urgent need to further our understanding of NE in order to develop novel interventions ^16^. Differences in the nature and timing of brain insult may occur due to variance in care settings and related exposures, supporting the need to focus on understanding the aetiology, nature and timing of brain injury in LMIC settings. This pilot feasibility study will be one of the first in a sub-Saharan African country, outside South Africa, to use sophisticated neurological investigation techniques such as continuous video EEG and MRI/MRS, providing vital data on the aetiology, nature, timing of brain injury and outcomes in NE in a sub-Saharan African population. The findings from the proposed work will be used to support the establishment of future NE research infrastructure in Uganda encompassing both observational and interventional research. The Baby BRAiN study aims to provide data relevant to promoting our understanding of NE in LIC settings in Africa, and contribute to the development of innovative strategies for NE globally.

### Limitations of the data

As only neonates admitted to the KNRH neonatal unit will be screened for eligibility, our study will not capture those who die immediately after birth or are stillborn at term as a result of severe NE. Due to the gestational age and birthweight restriction, we will not be able to comment on preterm brain injury, or term neonates who have severe intra-uterine growth restriction (IUGR). Evidence of perinatal asphyxia in the eligibility criteria will be based clinically on the need for resuscitation after birth and Apgar score, as routine fetal monitoring and blood gas measurement are not routinely performed. The extended recruitment criteria of 48 hours may capture neonates with a wider aetiology than hypoxia-ischaemia. MRI scans will not be available for neonates that die before day 10–14, those that are not clinically stable enough to be transferred for scanning, or for those born during the first Covid-19 related lockdown. As the imaging centre is located remotely, only those that are clinically stable for transfer will be able to attend; this may exclude those with the most severe NE. Whilst every attempt will be made to ensure that neurodevelopmental assessments are conducted blind to early clinical data this may not be completely assured due to overlap between staff performing neonatal care and follow-up assessments. As the study site is a national referral hospital in an urban location, the findings may not be representative of children with NE in Uganda more broadly.

### Potential challenges

The consenting process may be challenging due to the need to recruit soon after birth, the involvement of unwell neonates and potentially unwell mothers, and for those with lower parental literacy rates. Up to 48 hours after birth will be allowed for parents to make a decision on participation in the study; where mothers are unavailable or too unwell to consent, fathers will be approached, and a continuous consent approach will be followed ^51, 52^.

The study requires specialised and often expensive equipment, which may present challenges with procurement, maintenance and repair. Further, many of the study procedures require intensive monitoring which may be challenging in this low-resource setting. Parents may be concerned about the technology, particularly EEG and MRI; however, we have not experienced this in previous studies in Uganda and Ghana. Every effort will be made to explain (and where appropriate, demonstrate) the procedures in the local language and without medical jargon.

### Data availability

No data are associated with this article.

### Reporting guideline

The STROBE guidelines for reporting observational (cohort) research were used in preparing this protocol manuscript.
