# Supplementary material for: Protocol for the Birth Asphyxia in African Newborns (Baby BRAiN) Study: a Neonatal Encephalopathy Feasibility Cohort Study
Source: Gates Open Res. 2022 Mar 3;6:10. [Version 1] doi: 10.12688/gatesopenres.13557.1 (PMC9110736; doi:10.12688/gatesopenres.13557.1)
Supplement: Peer review history

# Peer review: Reviewer response for version 1

**DOI:** 10.21956/gatesopenres.14824.r31906

**Published:** 2022-05-16

**Authors:** Srinivas Kota

**Affiliations:** 1 Abbott Diabetic Care, Maidenhead, UK

This prospective study aims to establish a pilot neonatal encephalopathy cohort based at Kawempe National Referral Hospital to investigate clinical course, nature and timing of perinatal injury and associations with adverse outcomes, and in addition to multiple neurodevelopmental assessments before 24 months of age. 

 In the data analysis, it was mentioned that the electrographic seizure burden and seizures were recognized by bedside staff. What is the feasibility of this by bedside staff? Was there a dedicated staff member for each neonate for 24 hours a day?

 Attrition could be a major challenge for follow-up indicated in table 1. The authors could mention the approaches that they are considering to reduce attrition rate for follow-ups. Looking forward to learning findings from the current study. 

 I believe the recruitment might have been completed by now for this study. The authors could plan for a study involving neonates with mild injury in the future. Chalak *et al.* (2019 ^1^) observed that total sarnat score >= 5 performed within six hours of life detected future disability.

Is the study design appropriate for the research question?

Yes

Is the rationale for, and objectives of, the study clearly described?

Yes

Are sufficient details of the methods provided to allow replication by others?

Yes

Are the datasets clearly presented in a useable and accessible format?

Not applicable

Reviewer Expertise:

computational neuroscience, physiological signal processing, neonatal neurology, biomedical engineering, EEG/ERP

I confirm that I have read this submission and believe that I have an appropriate level of expertise to confirm that it is of an acceptable scientific standard.

## References

[1] : A Total Sarnat Score in Mild Hypoxic-ischemic Encephalopathy Can Detect Infants at Higher Risk of Disability. *J Pediatr* .214: 10.1016/j.jpeds.2019.06.026 217-221.e1 10.1016/j.jpeds.2019.06.026 31301853

# Peer review: Reviewer response for version 1

**DOI:** 10.21956/gatesopenres.14824.r31822

**Published:** 2022-03-18

**Authors:** Linda S. De Vries

**Affiliations:** 1 Department of Neonatology, University Medical Center, Utrecht University, Utrecht, The Netherlands

This is a prospective pilot feasibility cohort study of neonates with NE conducted at Kawempe National Referral Hospital, Kampala, Uganda. Neurological investigations include continuous video electroencephalography (EEG) (days 1-4), serial cranial ultrasound imaging, and neonatal brain Magnetic Resonance Imaging and Spectroscopy (MRI/ MRS) (day 10-14). Neurodevelopmental follow-up will be continued to 18-24 months of age.

 The project is certainly of importance and performed by a group with considerable expertise in all domains. The project is written like it will be started soon, but in fact it is almost completed with the last infants seen at follow-up next month. So any suggestions for the protocol are no longer helpful. The number collected (50) is maybe not large enough to answer the questions raised.

 MRI is performed outside the DWI window for several reasons. As they mention in the discussion, only those who did survive and were stable enough to go to the MRI unit were scanned. This may result in a selection bias.

 I am not sure why they used 5 on the Thompson score as a cut-off rather than the more commonly used 7, maybe to not restrict the population too much.

 I am also not sure why they used diazepam rather than midazolam as a second line ASM, maybe because of easier access.

 I wonder whether the clinicians also had access to 2 channels of aEEG, as this will be easier to interpret for the neonatologist working in the unit.

 As there is a considerable risk of antenatal onset of the infant’s problems, a very early ultrasound <6 hrs) will be helpful to support a potential antenatal onset. However recruitment allowed enrolment up until 48 hours which will make assessment of timing of the injury very difficult except for infants with established antenatal injury. In the section potential challenges, they say that parents were allowed to make a decision on participation up to 48 hours after birth. Did they collect data before consent was given?

Is the study design appropriate for the research question?

Yes

Is the rationale for, and objectives of, the study clearly described?

Yes

Are sufficient details of the methods provided to allow replication by others?

Yes

Are the datasets clearly presented in a useable and accessible format?

Yes

Reviewer Expertise:

neonatal neurology

I confirm that I have read this submission and believe that I have an appropriate level of expertise to confirm that it is of an acceptable scientific standard.
